# Accumulation of Protease Mutations among Patients Failing Second-Line Antiretroviral Therapy and Response to Salvage Therapy in Nigeria

**DOI:** 10.1371/journal.pone.0073582

**Published:** 2013-09-17

**Authors:** Holly E. Rawizza, Beth Chaplin, Seema T. Meloni, Kristin M. Darin, Oluremi Olaitan, Kimberly K. Scarsi, Chika K. Onwuamah, Rosemary A. Audu, Philippe R. Chebu, Godwin E. Imade, Prosper Okonkwo, Phyllis J. Kanki

**Affiliations:** 1 Brigham and Women’s Hospital, Boston, Massachusetts, United States of America; 2 Harvard School of Public Health, Boston, Massachusetts, United States of America; 3 Northwestern University Feinberg School of Medicine, Chicago, Illinois, United States of America; 4 AIDS Prevention Initiative in Nigeria, Abuja, Nigeria; 5 Nigerian Institute of Medical Research, Lagos, Nigeria; 6 Jos University Teaching Hospital, Jos, Nigeria; University of Pittsburgh, United States of America

## Abstract

**Background:**

To date, antiretroviral therapy (ART) guidelines and programs in resource-limited settings (RLS) have focused on 1^st^- and 2^nd^-line (2 L) therapy. As programs approach a decade of implementation, policy regarding access to 3^rd^-line (3 L) ART is needed. We aimed to examine the impact of maintaining patients on failing 2 L ART on the accumulation of protease (PR) mutations.

**Methods and Findings:**

From 2004–2011, the Harvard/APIN PEPFAR Program provided ART to >100,000 people in Nigeria. Genotypic resistance testing was performed on a subset of patients experiencing 2 L failure, defined as 2 consecutive viral loads (VL)>1000 copies/mL after ≥6 months on 2 L. Of 6714 patients who received protease inhibitor (PI)-based ART, 673 (10.0%) met virologic failure criteria. Genotypes were performed on 61 samples. Patients on non-suppressive 2 L therapy for <12 months prior to genotyping had a median of 2 (IQR: 0–5) International AIDS Society (IAS) PR mutations compared with 5 (IQR: 0–6) among patients failing for >24 months. Patients developed a median of 0.6 (IQR: 0–1.4) IAS PR mutations per 6 months on failing 2 L therapy. In 38% of failing patients no PR mutations were present. For patients failing >24 months, high- or intermediate-level resistance to lopinavir and atazanavir was present in 63%, with 5% to darunavir.

**Conclusions:**

This is the first report assessing the impact of duration of non-suppressive 2 L therapy on the accumulation of PR resistance in a RLS. This information provides insight into the resistance cost of failing to switch non-suppressive 2 L regimens and highlights the issue of 3 L access.

## Introduction

Over 8 million individuals living with HIV in low- and middle-income countries are receiving antiretroviral therapy (ART) [1]. As large-scale ART programs in resource-limited settings (RLS) approach a decade of implementation, the 2010 World Health Organization (WHO) public health guidelines for the first time raised the issue of access to third-line ART and called on national programs to delineate policies [2].

As with access pricing for first-line (1 L) and second-line (2 L) ART, the cost of third-line (3 L) ART has decreased nearly ten-fold in the past several years [3]. However, it is yet to be determined whether there is a significant need for 3 L ART among patients in RLS. Published rates of 2 L failure vary, ranging from as low as 14.3% at 24 months follow-up [4] to as high as 50% of patients with confirmed virologic failure [5]–[10]. A number of studies suggest that the majority of patients who fail 2 L ART do so with minimal drug resistance, suggesting poor adherence rather than resistance as the cause of failure. In one study, wild-type virus was present at the time of failure in up to 67% of patients with no major protease (PR) mutations identified [11]–[12]. However, studies vary widely, with 28% of patients harboring at least one PR mutation after 2 L failure in a study from Mali [13], in contrast to 73% of 2 L failures in a cohort from India [14].

The variability is not surprising given differences in duration of failure prior to genotype testing among the studies. To date, studies have been limited by either a short or unknown duration of follow-up on failing 2 L regimens. Although resistance to non-nucleoside reverse transcriptase inhibitors (NNRTI) develops rapidly and for those NNRTIs utilized in 1 L regimens only requires a single point mutation [15], [16], resistance to protease inhibitors (PI) requires sequential accumulation of mutations in the setting of ongoing exposure to non-suppressive PI-based ART [17].

Additionally, after failure of 1 L ART, 2 L options may be limited in settings where nucleoside reverse transcriptase inhibitors (NRTI) are restricted [18]. In one study, failure of a PI-based 2 L regimen was more closely associated with failure to change the NRTI backbone rather than level of adherence [19]. Decreased susceptibility to the NRTI component of a regimen may, therefore, increase the risk of developing PI resistance [20], [21].

Here, we report on rates of 2 L treatment failure from a large ART program in Nigeria, with a focus on the accumulation of PR mutations according to time on failing 2 L ART among a subset of patients who underwent genotype testing. Additionally, we present the first outcome data from a small cohort of patients receiving 3 L ART in Nigeria. The challenge remains to determine how, or perhaps whether, policy makers, implementing partners, national governments, and pharmaceutical companies will address the inevitable need for 3 L therapy among patients living in RLS.

## Methods

### Study Setting

The Harvard/AIDS Prevention Initiative in Nigeria (APIN) President’s Emergency Plan for AIDS Relief (PEPFAR) program provided HIV treatment to over 100,000 individuals at 32 clinical sites in Nigeria from 2004–2011. Consistent with the Nigerian national [22] and WHO treatment guidelines [2], [23], 1 L ART consisted of one NNRTI plus two NRTIs. Second-line ART consisted of a ritonavir-boosted PI plus 2 NRTIs (selected to differ from those used in 1 L regimen). In this retrospective analysis, the study cohort consisted of HIV-infected adults for whom 2 L failure was confirmed and genotype testing was performed.

### Ethical Considerations

All patients enrolled in the Harvard/APIN PEPFAR program provided written consent for care; data for those that also consented for use of their information in future analyses were evaluated. The treatment protocol and written consent were approved by the institutional review boards (IRBs) of the Harvard School of Public Health and by each of the affiliated treatment sites. All program-affiliated Nigerian IRBs are registered with the U.S. Federalwide Assurance.

### Definition of Treatment Failure and Selection Criteria for Genotyping

Based on the program protocol, virologic failure was defined as two consecutive viral load (VL) measurements >1000 copies/mL (cpm) after at least 6 months of ART. For patients failing a 2 L regimen, genotypic resistance testing was considered if adequate adherence was demonstrated. Adequacy of adherence was determined by individual clinicians and typically required >95% adherence to the pharmacy pick-up schedule during the 3–6 months prior to the last detectable VL measurement.

The *duration of 2* *L failure* was based on either the time from 2 L initiation to genotype testing, if the patient never achieved virologic suppression, or the time from last suppressed VL to genotype testing for those who initially achieved suppression on 2 L. The overall duration of 2 L ART was also determined, representing time from 2 L initiation to genotype testing in this study.

### Adherence Assessment

Adherence was estimated using pharmacy pickup data. Patients were given either a 30- or 60-day supply of ART, and average adherence was calculated as the proportion of time the patient had pills available to them from 2 L initiation to the date of genotyping. For instance, if a patient presented early to collect the prescription one month, this excess pill supply was accounted for during subsequent pick-ups.

### Data Collection

Patient data were collected on paper clinic forms and prescription pads. The data were then entered into a customized electronic record database by trained data staff at each site. Baseline evaluations included medical history, physical examination, WHO clinical staging, CD4+ cell count, plasma VL, complete blood counts, and chemistries. These same clinical and laboratory evaluations were performed 3 and 6 months after ART initiation, then approximately every 6 months thereafter unless symptoms required more frequent monitoring. Additionally, pharmacists entered dispensing information directly into a customized electronic database that mirrors the prescription pad, providing real-time visual confirmation of patient pharmacy refill history.

### Laboratory Analysis

All laboratory tests were performed in Nigeria by trained laboratory staff at each clinic facility. CD4+ cell count measurement was performed using laser-based CD4 T-lymphocyte enumeration (Cyflow, Partec, Germany). Plasma HIV-1 RNA VL determination was performed using the Roche Cobas Amplicor Monitor assay, version 1.5 (Roche Diagnostics, New Jersey, USA). All laboratories participate in regular external quality-control programs for HIV diagnosis, CD4+ cell enumeration, and plasma VL estimation.

### Genotype Testing

Starting in 2008, the Harvard/APIN PEPFAR program began developing the laboratory infrastructure to perform HIV genotype testing at three sites in Nigeria including, Jos University Teaching Hospital in Jos, Plateau State, University College Hospital in Ibadan, Oyo State, and the Nigerian Institute of Medical Research in Lagos, Lagos State. Drug resistance genotyping was performed on a 3130 ABI sequencer (Applied Biosystems, Foster City, CA, USA) using the Viroseq HIV-1 Genotyping system (Abbott Molecular, Des Plaines, IL, USA) to reverse transcribe and amplify 297 bases of the PR and 1005 bases of reverse transcriptase (RT) genes. Sequence was edited and compared to an HXB2 subtype B reference with the manufacturer’s software to generate lists of mutations and polymorphisms. Sequences were aligned in ClustalW (Belfield, Dublin, Ireland) along with reference sequences from the Los Alamos repository [24] and neighbor-joining trees were used to classify them by subtype.

The clinical significance of identified drug resistance mutations was determined using the Stanford HIV Drug Resistance Database and the 2011 version of the International AIDS Society (IAS) drug resistance update [25], [26]. The estimated rate of accumulation of PR mutations was calculated by dividing the total number of major and minor IAS PR mutations by the duration of 2 L failure.

Genotype sensitivity scores (GSS) for the prescribed regimen at the time of 2 L failure were calculated based on the five Stanford HIVdb resistance categories: susceptible or complete activity, potential low-level resistance or good activity, low-level resistance or partial activity, intermediate-level resistance or scarce activity, high-level resistance or no activity corresponding with scores of 1.00, 0.75, 0.50, 0.25, and 0.00, respectively [25], [27].

### Third-line Treatment

A small number of patients with 2 L failure and evidence of extensive PR resistance were initiated on 3 L ART. Appropriate 3 L regimens were determined based on resistance profiles and the availability of a limited supply of 3 L drugs, most commonly consisting of a combination of darunavir/ritonavir (DRV/r), raltegravir (RAL), recycled nucleosides, and, in cases where indicated, etravirine (ETR).

### Statistical Analyses

Statistical analyses were performed using Stata version 10.1 (StataCorp, College Station, TX). For continuous variables, the nonparametric Wilcoxon test was used due to small sample sizes and lack of normally distributed data. The Fisher’s exact test was used for categorical variables. Variables at the p<0.05 were considered statistically significant.

## Results

### Study Population

Since the program began in 2004, over 100,000 patients have initiated ART within the Harvard/APIN PEPFAR program. Of these, 6714 (approximately 7%) have received PI-based 2 L therapy. Of patients receiving 2 L ART, 673 (10.0%) met the program-defined virologic failure criteria of 2 consecutive VLs>1000 cpm. Of note, if a VL failure definition of confirmed VL>5000 cpm, as is consistent with the 2010 WHO guidelines [1], had been utilized, the number of patients characterized as failures would have decreased to 354 individuals (5.3%; 52.6% of 673 failures), with the diagnosis of failure being delayed a mean of 7.6 months in 50 of those patients.

Genotypes were performed on 61 patient samples. In general, patients selected for genotyping were more immunocompromised, but more adherent, and had been on 2 L ART for longer than those who were not selected. Comparing the 61 patients for whom genotypes were performed to the 612 patients who failed, but were not selected for genotype testing, the median CD4+ cell count was 215 cells/mm^3^ (IQR: 116–291) versus 265 cells/mm^3^ (IQR: 158–429; p = 0.006) and the median 2 L adherence was 93% versus 88% (p = 0.008), respectively. The total duration of 2 L ART was 3.9 years (IQR: 2.9–4.8) versus 3.2 years (IQR: 2.4–4.2; p = 0.003), respectively. Total antiretroviral exposure was similar for the patients who underwent genotype testing versus those who did not, with the median number of prior antiretroviral drugs being 6 (IQR: 5–9) versus 6 (IQR: 5–6).

Results from the 61 patients who underwent genotype testing were stratified by time on failing 2 L regimen (0–12 months (n = 13), 13–24 months (n = 29), and >24 months (n = 19); Table 1). All patients were receiving lopinavir/ritonavir (LPV/r) at the time of 2 L failure, although prior exposure to saquinavir, indinavir, or both occurred in 10% (n = 6), 7% (n = 4), and 2% (n = 1), respectively. Overall, the median duration on 1 L ART prior to 2 L switch was 25 months (IQR: 16–34 months) and median patient age at time of 2 L initiation was 40 years (IQR: 32–44 years). The aggregate patient characteristics at the time of 2 L failure included: median CD4+ cell count of 215 cells/mm^3^, median VL of 13,835 cpm, median duration of 2 L failure of 19 months (IQR: 13–28 months), and 44% of the cohort was female.

**Table 1:** Patient Characteristics by Duration of Second-Line Treatment Failure.

	Duration of Second-Line Failure^a^			
	0–12 months	13–24 months	>24 months	All Patients
Characteristic	(n = 13)	(n = 29)	(n = 19)	(n = 61)
**Age (years)**	42 (32–44)^b^	38 (33–42)	40 (32–49)	40 (32–44)
**Gender, female (%)**	38%	35%	63%	44%
**# ARVs previously used, median (range)**	6 (4–7)	6 (4–8)	6 (5–9)	6 (5–9)
**Duration on 1 L (months)**	22 (19–36)	28 (21–36)	17 (15–26)	25 (16–34)
**Duration on 2 L (months)** c	13 (11–38)	20 (18–26)	39 (34–47)	26 (18–38)
**Duration of 2 L Failure (months)** a	11 (6–11)	17 (16–20)	34 (30–42)	19 (13–28)
**Adherence to 2 L (%), median**	88 (77–100)	96 (85–100)	93 (81–100)	93 (81–100)
**CD4 at 2 L failure (cells/mm^3^)**	208 (108–269)	225 (118–296)	205 (108–304)	215 (116–291)
**VL at 2 L failure (copies/mL)**	29,068 (7,918–130,037)	7,830 (3,264–22,696)	75,600 (1,989–195,879)	13,835 (2,954–99,344)

a Time from 2 L initiation, or last suppressed VL on 2 L, to genotype sample.

b Numbers represent: Median (IQR) unless otherwise indicated.

c Time from 2 L initiation to genotype sample.

### Accumulation of Protease Mutations

Among patients who had been failing 2 L ART for >24 months, the median number of IAS PR mutations was 5 (IQR: 0–6) compared with 1 (IQR: 0–4) for those failing 13–24 months, and 2 (IQR: 0–5) for those failing for ≤12 months. Using these cross-sectional data, we estimated that patients developed a median of 0.6 (IQR: 0–1.3) IAS PR mutations for every 6 months on a failing 2 L regimen (Table 2).

**Table 2:** Accumulation of Protease Mutations According to Duration of Second-Line Treatment Failure.

	Duration of Second-Line Failure			
	0–12 months	13–24 months	>24 months	All Patients
Characteristic	(n = 13)	(n = 29)	(n = 19)	(n = 61)
**Patients with ≥1 IAS PR mutation, n (%)**	8 (62%)	16 (55%)	14 (74%)	38 (62%)
**# Protease mutations, median (IQR)**	2 (0–5)	1 (0–4)	5 (0–6)	2 (0–5)
** Major PR mutations (IAS)**	2 (0–3)	0 (0–3)	3 (0–4)	1 (0–3)
** Minor PR mutations**	0 (0–1)	0 (0–2)	2 (0–3)	1 (0–2)
**Patients with no PR mutations, n (%)**	5 (38%)	13 (45%)	5 (26%)	23 (38%)
**High- or Intermediate-level PI Resistance, n (%)**				
** Lopinavir (LPV/r)**	7 (54%)	11 (38%)	12 (63%)	30 (49%)
** Atazanavir (ATV/r)**	5 (38%)	11 (38%)	12 (63%)	28 (46%)
** Darunavir (DRV/r)**	0 (0%)	4 (14%)	1 (5%)	5 (8%)
**Genotypic Sensitive Score**	1 (0.25–3)	1.25 (0.5–4)	0.5 (0.25–1.5)	1.25 (0.25–3)
**# PR mutations for every 6 mo. on Failing 2** **L Regimen (n = 61)**	2.0 (0–3.0)	0.3 (0–1.4)	0.8 (0.1–1.1)	0.6 (0–1.4)
**# PR mutations for every 6 mo. on Failing 2** **L Regimen (n = 38, patients with no PR mutations excluded)**	(n = 8)	(n = 16)	(n = 14)	(n = 38)
	2.9 (2.3–3.5)	1.3 (0.6–2.4)	1.0 (0.7–1.2)	1.2 (0.8–2.4)

Although 62% of patients had one or more PR mutations, wild-type virus (no RT or PR mutations) was present in 18% of patients with genotypes, while 20% of failing patients had at least one RT mutation but no PR mutations. Since the acquisition of PR mutations is a progressive process [17], [28], [29], where patients with established PR resistance are more likely to acquire additional PI mutations, we also examined the rate of accumulation among the 38 patients with at least one major or minor PI mutation. Among this subset of patients, the median number of IAS PR mutations for every 6 months on a failing 2 L regimen increased to 1.2 (IQR: 0.8–2.4).

For patients failing >24 months, high- or intermediate-level resistance to LPV/r and atazanavir/ritonavir (ATV/r) was present in 63% of patients and to DRV/r in 5% of patients. Among the 61 patients, the median GSS for the 2 L regimen was 1.25 (IQR: 0.25–3) (Table 2).

### Distribution of Drug Resistance Mutations

**Figure 1:**
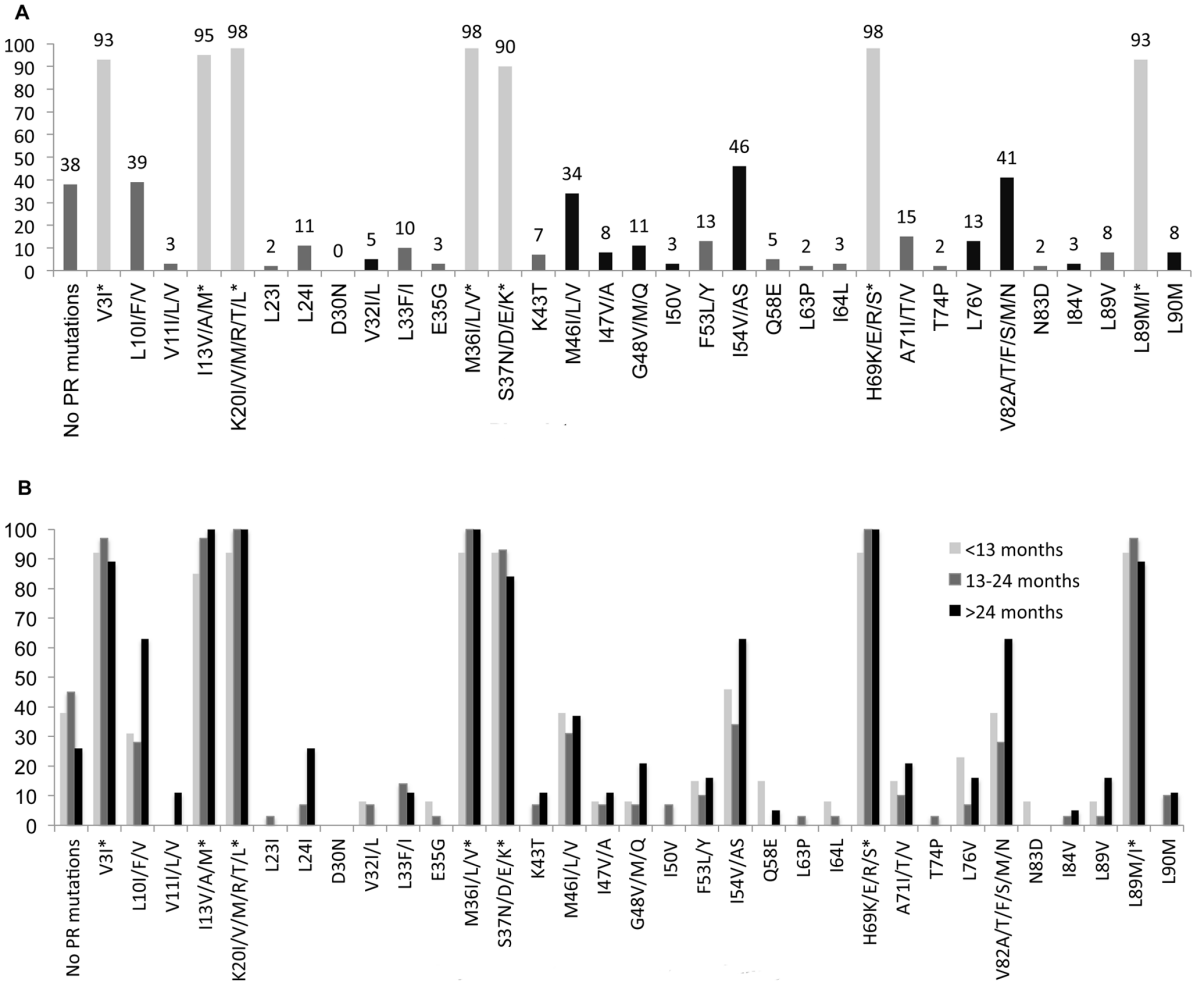
Frequency of protease (PR) mutations among 61 patients failing second-line ART. (A) Total prevalence of PR mutations. Major PR mutations are in black, minor PR mutations are in dark grey, and polymorphisms (mutation position labeled with *) are in light grey. (B) Prevalence of PR mutations stratified by time on failing second-line regimen.

The distribution of HIV-1 subtypes included: CRF02_AG (41%, n = 25), G-prime (34%, n = 21), CRF06_cpx (7%, n = 4), G (5%, n = 3), one patient each with subtype C, A3, and CRF22; the subtype was unknown in 8% (n = 5). The most common major PR mutations included: I54V/A/S (n = 28, 46%), V82A/F/T/S/M (n = 25, 41%), M46I/L (n = 21, 34%), L76V (n = 8, 13%), and G48V/M/Q (n = 7, 11%) (Figure 1). A number of PR polymorphisms common among non-subtype B virus were also identified, consistent with previous studies [16], [30]. Those polymorphisms present in >90% of patients in this cohort included: V3I, I13V/A/M, K20I/V/M/R/T/L, M36I/L/V, S37N/D/E/K, H69K/E/R/S and L89M/I (Figure 1).

**Figure 2:**
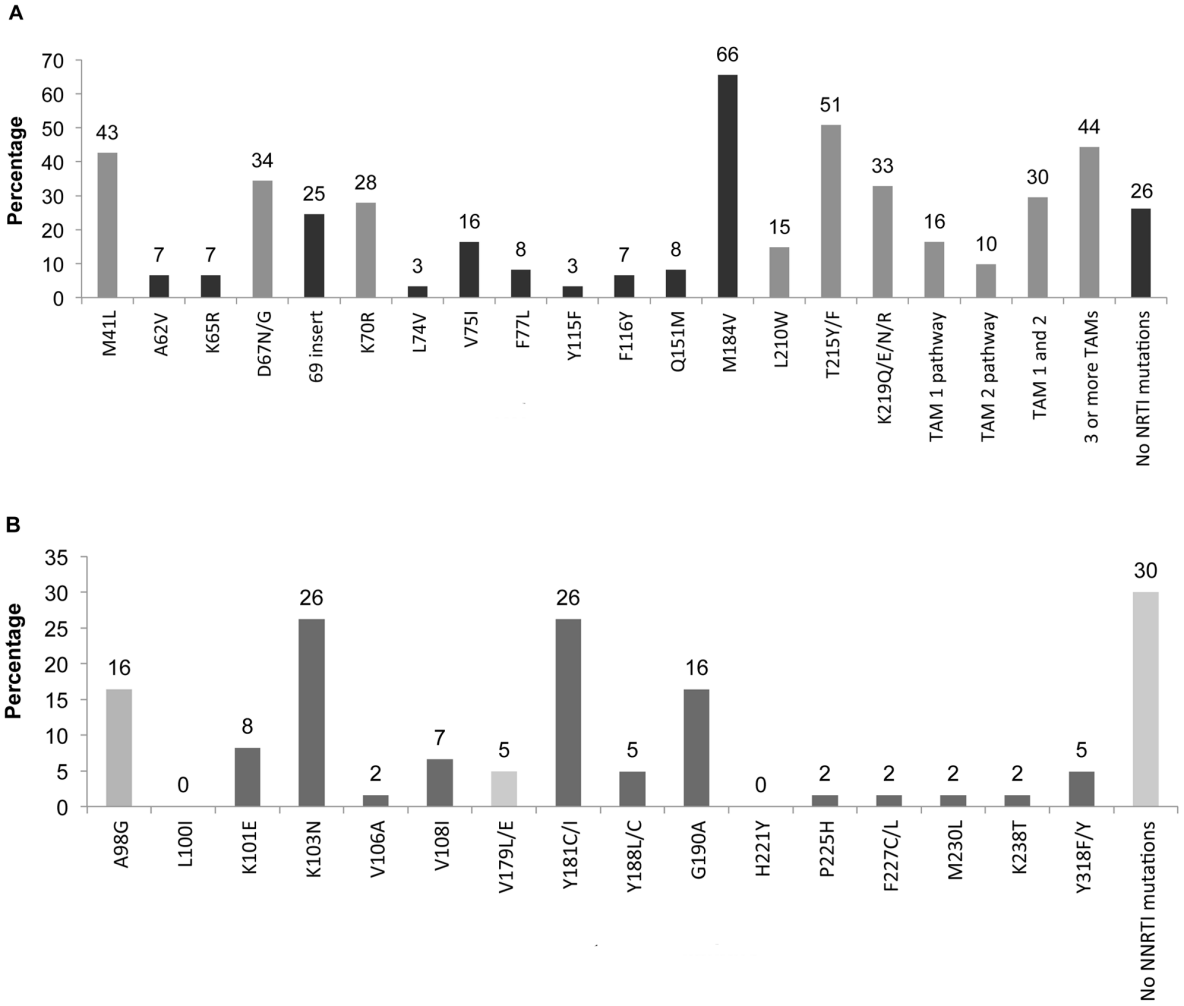
Prevalence of reverse transcriptase mutations. (A) NRTI resistance mutations; thymidine analog mutations (TAMs) are in light grey. (B) NNRTI resistance mutations; minor mutations indicated by light grey.

At least one thymidine analog mutation (TAM) was present in 56% of patients at the time of genotype testing, with 44% (27 patients) having 3 or more TAMs (Figure 2). Among the 38 patients with 1 or more IAS PR mutations, all but 2 patients had at least one NRTI mutation. The prevalence of NNRTI mutations likely underestimate archived resistance given that patients had been off NNRTI-based therapy for varying durations. However, 28% (17 of 61) had high- or intermediate-level resistance to ETR at the time of genotype.

### Third -Line ART Program Experience

Among this cohort of 61 patients, 11 patients with documented high-level resistance to LPV/r initiated 3 L ART (Table 3). All 11 patients initiated a 3 L regimen containing DRV/r and RAL, with 6 patients also receiving ETR and 8 patients receiving recycled NRTIs, based on drug resistance profiles. At a median of 31 months after initiating 3 L ART (IQR: 29–39 months), 9 of 11 patients remained virologically suppressed (VL<400 cpm). One patient died within 6 months of initiating 3 L ART due to complications of tuberculosis, sepsis, and renal failure. This corresponds with a suppression rate of 82% after a median of 2.6 years on 3 L ART.

**Table 3:** Characteristics for Patients who Initiated Third-Line ART.

	Median (IQR)^c^
**Characteristic**	**(n = 11)**
**Age at 3** **L Initiation (years)**	48 (45–52)
**Gender, female (%)**	36%
**# ARVs previously used, median (range)**	6 (4–7)
**Duration on 1** **L, months**	23 (12–33)
**Duration on 2** **L, months**	28 (16–36)
**Duration of 2** **L Failure** d **, months**	20 (14–35)
**Adherence to 2** **L (%), median**	89 (76–100)
**CD4 at 3** **L switch (cells/mm^3^)**	75 (41–120)
**VL at 3** **L switch (copies/mL)**	111,749 (14807–482,472)
**Total # of IAS Protease mutations**	6 (6–7.5)
**Genotypic Sensitive Score**	0.25 (0.125–0.25)

c Results represent median (IQR) except where indicated.

d Time from 2 L initiation, or last suppressed VL on 2 L, to genotype sample.

## Discussion

This is one of the first reports to evaluate the rate of accumulation of PR mutations following 2 L failure in a RLS. Among patients failing 2 L ART for over 24 months, almost two-thirds developed regimen-compromising PR resistance with a median of five IAS PR mutations. On average, we estimated that patients developed 0.6 PR mutations for every 6 months they were maintained on a failing 2 L regimen.

A number of prior studies from RLS have reported low rates of PR resistance after 2 L failure [11], [31], [32], often attributing this finding to poor medication adherence. Although failure of PI-based ART without evidence of drug resistance is a well-documented phenomenon, and may be attributed to the relatively narrower range of adherence levels at which PI resistance develops [17], [33], these findings may be partially attributed to the short duration of 2 L ART exposure in these studies. In contrast, over 60% of patients in this study had one or more IAS PR mutation, placing them at increased risk of accumulating additional PI mutations.

Although the importance of intensified adherence counseling within programs cannot be overemphasized, for some patients, maintaining a failing 2 L ART regimen will result in increasingly resistant virus. This study was not intended to evaluate overall resistance among patients with 2 L failure, rather to assess the prevalence of PR mutations among patients identified by clinic-based practitioners as experiencing virologic failure in the setting of good adherence. The challenge in RLS remains how to determine which patients are failing due to non-adherence versus the presence of drug resistance mutations.

This analysis documented that over two-thirds of patients developed PR mutations at positions 46, 54, or 82, which have been associated with the rapid development of lopinavir resistance in prior studies [28]. Given the known cumulative nature of PR resistance [17], [28], one would expect increasing numbers of PR mutations over time, as was demonstrated in this study. Adding to the concern of suboptimal treatment, 44% of patients in this study had 3 or more TAMs and the median GSS at genotype testing was 1.25 (IQR: 0.25–3). Among the 32 patients with full susceptibility or only low-level LPV/r resistance, over one-third (11 of 32 patients) had significantly reduced activity of the NRTI backbone, suggesting that the patients were effectively receiving LPV/r monotherapy. LPV/r monotherapy has been shown in a number of studies to be suboptimal [34]–[36], and may increase the risk for resistance to PIs [37].

The majority of patients had preserved susceptibility to DRV/r, suggesting that 3 L therapy may be an option; however, 28% (17 of 61) had high- or intermediate-level resistance to ETR. As access pricing for 3 L ART continues to decline, with current estimates for a regimen of DRV/r, ETR, RAL and recycled nucleosides being approximately $2500 (in 2012 US dollars) per patient per year [3], and longevity of ART programs increases, the national government policy on 3 L access becomes ever more critical. Cost-effectiveness evaluations of 1 L and 2 L ART in RLS have consistently shown that access to ART is highly cost-effective [38], [39]. Formal cost analyses that include access to 3 L ART are needed.

Several study limitations should be mentioned. First, data regarding the clinical applicability of resistance mutations among the multiple HIV-1 subtypes occurring in Nigeria are limited. Thus, clinical assessments in this study were based on the Stanford HIV Resistance Database, which primarily utilizes data from subtype B virus. Additionally, accumulation of resistance mutations over time was based on between patient comparisons. Further studies examining intra-patient development of drug resistance mutations over time would strengthen the determination of rates of accumulation of PR mutations. Furthermore, the relatively small sample size of 61 genotypes may limit broader interpretation as rates of resistance accumulation are sensitive to patient selection characteristics. The selection criteria for genotype testing also required high levels adherence prior to testing, thus this analysis was restricted to a somewhat more adherent patient population. In addition, duration of failure and timing of genotype assays were limited to those VL measurements collected as part of routine clinical care. However, patients in this cohort were monitored relatively frequently, with an average of one VL evaluation every 4.9 months. Finally, we utilized population genotyping, thus minor variants were potentially missed.

Finally, in this report we have also provided the first outcome data on a cohort of patients receiving 3 L ART in sub-Saharan Africa. Although the small cohort size limits wider assumptions of efficacy, the preliminary outcomes suggest that 3 L therapy can be effectively implemented in a RLS with excellent rates of virologic suppression.

In conclusion, this study shows that patients maintained on non-suppressive 2 L ART accumulate increasing numbers of PR mutations over time, potentially compromising future ART options. In this cohort, nearly two-thirds of patients failing for more than 24 months had high- or intermediate-level LPV/r resistance. As ART programs in RLS continue to mature, there will be an increasing number of patients in need of 3 L treatment. Development of policy by governments and their partners is urgently required to address this emerging need.

### Public Access

Available sequences were submitted to GenBank with the following accession numbers: KF241459KF241513.
